# Changes in energy metabolism and respiration in different tracheal narrowing in rats

**DOI:** 10.1038/s41598-021-98799-8

**Published:** 2021-09-27

**Authors:** Yael Segev, Haiat Nujedat, Eden Arazi, Mohammad H. Assadi, Ariel Tarasiuk

**Affiliations:** 1 Shraga Segal Department of Microbiology, Immunology, and Genetics, Ben-Gurion University of the Negev, P.O Box 105, Beer-Sheva, 84105 Israel; 2 Sleep-Wake Disorders Unit, Soroka University Medical Center, P.O. Box 151, Beer-Sheva, 84105 Israel; 3 Department of Physiology and Cell Biology, Faculty of Health Sciences, Ben-Gurion University of the Negev, P.O Box 105, Beer-Sheva, 84105 Israel

**Keywords:** Experimental models of disease, Physiology, Respiration

## Abstract

Why obstructive sleep apnea (OSA) treatment does not completely restore healthy metabolic physiology is unclear. In rats, the need for respiratory homeostasis maintenance following airway obstruction (AO) is associated with a loss of thermoregulation and abnormal metabolic physiology that persists following successful obstruction removal. Here, we explored the effect of two different types of tracheal narrowing, i.e., AO and mild airway obstruction (mAO), and its removal on respiratory homeostasis and metabolic physiology. We show that after ten weeks, mAO vs. AO consumes sufficient energy that is required to maintain respiratory homeostasis and thermoregulation. Obstruction removal was associated with largely irreversible increased feeding associated with elevated serum ghrelin, hypothalamic growth hormone secretagogue receptor 1a, and a phosphorylated Akt/Akt ratio, despite normalization of breathing and energy requirements. Our study supports the need for lifestyle eating behavior management, in addition to endocrine support, in order to attain healthy metabolic physiology in OSA patients.

## Introduction

Obstructive sleep apnea (OSA) is a disorder involving upper airway obstruction (AO) during sleep, and is associated with metabolic abnormalities^1,2^. It is not clear why treatment of OSA does not completely restore healthy metabolic physiology, but accelerates weight gain^2–4^ rather than promoting weight loss in most adult patients^5–9^. Earlier studies found that weight gain following OSA treatment was not associated with a change in exercise habits or sleep quality, or improved sleepiness^6^—highlighting the need for lifestyle modifications in order to prevent weight gain^6,8^. Healthy metabolic physiology depends on the basal metabolic rate, locomotion movements, energy intake, and food choices^8,10–12^. OSA may elevate energy expenditure by inducing disrupted sleep, the increased work of breathing, or sympathetic activity^8,10,13,14^.

The upper airway obstruction (AO) in rats mimics many of the characteristics of OSA in humans including fragmented sleep, poor growth, and increased feeding^15–17^. Although AO animals substantially increased their caloric intake, this condition was associated with a energy crisis, i.e., a “malnutrition” phenotype and the inability to thermoregulate^15,18,19^. This condition is associated with a persistent elevation in feeding and energy expenditure long after the tracheal obstruction is removed^19,20^. The stomach-derived hormone ghrelin drives feeding behavior via hypothalamic growth hormone secretagogue receptor 1a (GHSR1a) and up regulation phosphoinositide 3-kinases (PI3K)/Akt pathway. The PI3K/Akt pathway is required for normal metabolism and its imbalance leads to obesity, insulin resistance and type 2 diabetes^21–23^. It is possible that feeding hormones and imbalance of hypothalamic pathways participate in the persistent elevation of feeding behavior following obstruction removal (OR).

Previously, we found that increased airway resistance led to increased carbon dioxide production and increased ventilation to maintain respiratory homeostasis^18,20^. AMP-activated protein kinase (AMPK) is a major energy sensor that may become activated by hypercapnia, hypoxemia, or lactic acidosis^24–26^. Chronic hypercapnia can lead to AMPK-dependent loss of body weight gain and muscle protein wasting^24,25^.

Little is known about the effect of mild airway obstruction (mAO) and its removal on metabolic physiology. Here, we explored, for the first time, the effect of two different tracheal narrowing procedures, i.e., AO and mAO, and the removal of the obstruction on respiratory homeostasis and metabolic physiology. It is possible that mAO animals increases their caloric intake and compensates for the increased energy demand associated with the increased work of breathing, and maintaining healthy metabolic physiology. We hypothesized that mAO would lead to persistent elevation in feeding behavior that may not normalize long after OR and the restoration of respiration and energy expenditure compared to a control.

## Results

**Figure 1:**
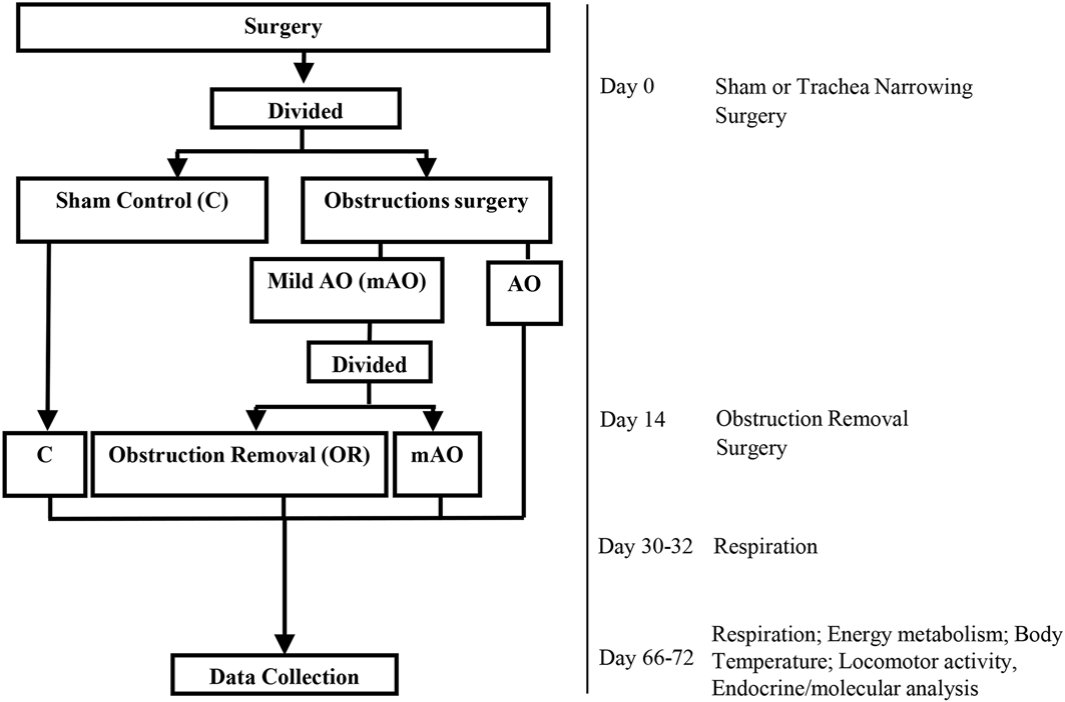
Flow diagram of study groups and time data collected.

**Figure 2:**
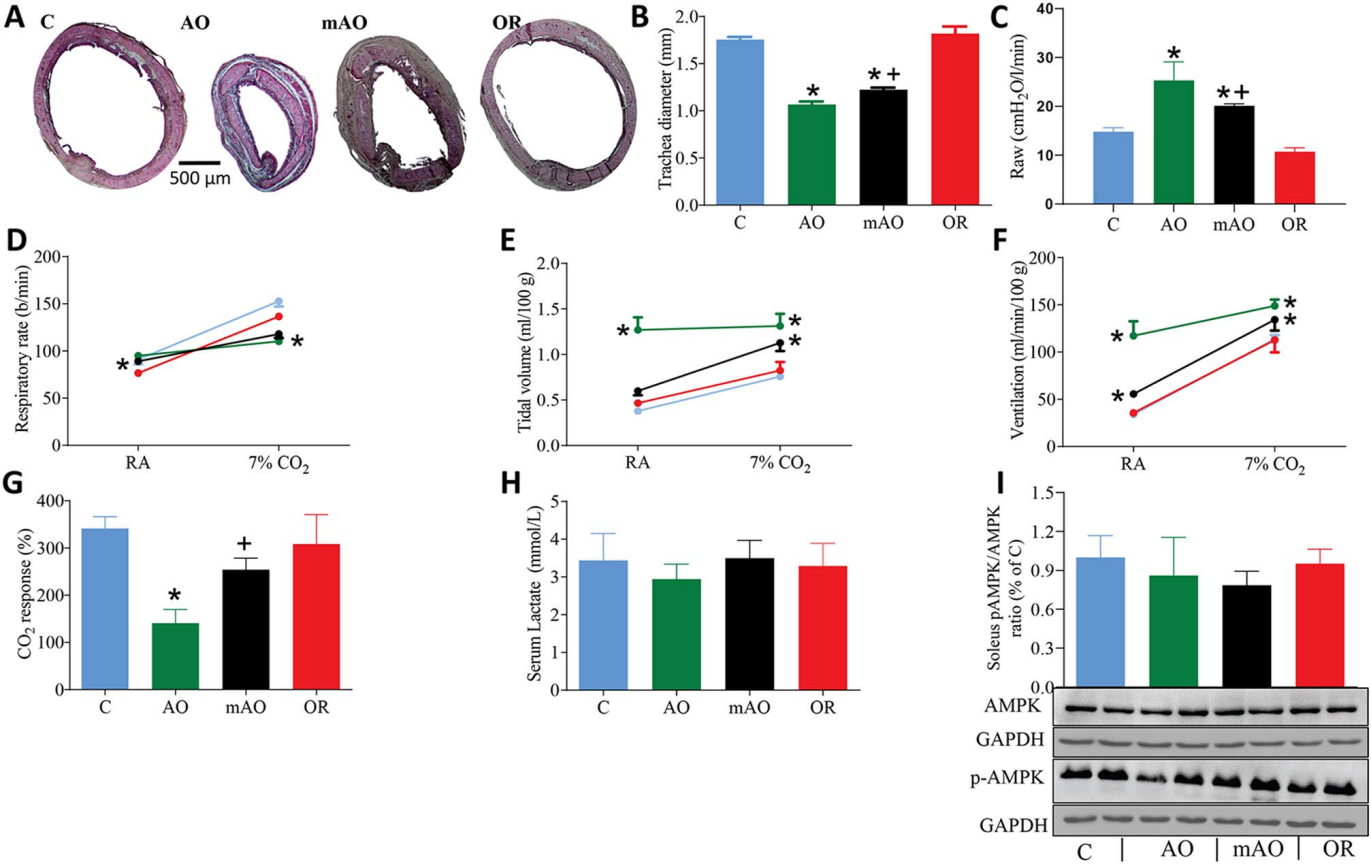
Respiratory activity: **(A)** representative image of tracheas; **(B)** trachea diameter; **(C)** airway resistance; **(D)** respiratory rate; **(E)** tidal volume; **(F)** minute ventilation; **(G) **CO_2_ response calculated as the percent change in minute ventilation from room-air breathing to 7% CO_2_ stimulation; **(H)** serum lactate level; **(I)** soleus p-AMPK/AMPK ratio. Raw—airway resistance; AMPK—5' AMP-activated protein kinase; blue—control (C); green—obstructive (AO); black—mild obstructive (mAO); red—obstruction removal (OR). Values are mean ± SEM. * *p* < 0.01, C vs. AO or mAO group. + *p* < 0.05, AO vs. mAO. In **(B,F–H)**, statistical differences were determined by a one-way ANOVA. In **(B–E)**, statistical differences were determined by a two-way ANOVA, followed by a post-hoc Student–Newman–Keuls test.

Figure 1 illustrates the time-line of data collection. During the observation period, all animals engaged in normal social activity. The trachea diameter was 1.81 ± 0.1 (mm), 1.04 ± 0.1 (mm) (*p* < 0.01), 1.19 ± 0.12 (*p* < 0.01), and 1.87 ± 0.11 (mm) (mean ± SD) (*p* = 0.7) for the control, AO, mAO, and OR groups, respectively (Fig. 2A,B). Following tracheal obstruction, airway resistance increased by 71% and 35% (*p* < 0.01) in the AO and mAO groups relative to the control group, respectively (Fig. 2C, Table 1). Following OR, trachea diameter and airway resistance were restored to control values. Figure 2D–F summarizes the effect of mAO and its removal on respiration. Increased airway resistance was associated with 294% and 64% elevation of ventilation during room air breathing in the AO and mAO groups, respectively (*p* < 0.01, Fig. 2D–F, Table 1). CO_2_ sensitivity decreased by 59% and 25.5% in the AO and mAO groups, respectively (*p* < 0.01, Fig. 2G, Table 1). Following OR, both ventilation and CO_2_ sensitivity were similar to controls. No significant changes were found in arterial blood gases (Supplementary Table S1), serum lactate (Fig. 2H, Supplementary Fig. S1), or soleus muscle pAMPK/AMPK ratio (Fig. 2I).

**Table 1:** Respiratory data.

	Group	Days after surgery	
		35	66–72
∆Pes (cmH_2_O)	Control		10.5 ± 0.6
	AO		18.3 ± 1.5*
	mAO		15.0 ± 1.0*^+^
	OR		9.3 ± 0.4
Raw (cmH_2_O/l/min)	Control		14.8 ± 0.8
	AO		25.3 ± 3.8*
	mAO		20.1 ± 0.4*^+^
	OR		10.7 ± 0.8
Room air ventilation (ml/min/100 g)	Control	52.1 ± 2.2	33.9 ± 1.7
	AO	109.8 ± 20.1*	133.5 ± 15.2*
	mAO	58.0 ± 2.8^+^	55.6 ± 3.6*^+^
	OR	58.9 ± 3.7	35.9 ± 2.0
7% CO_2_ ventilation (ml/min/100 g)	Control	161.1 ± 7.8	113.9 ± 4.2
	AO	175.0 ± 7.7	146.9 ± 10.7*
	mAO	180.7 ± 9.3	140.06 ± 9.3
	OR	157.1 ± 6.2	110.8 ± 11.4
CO_2_ response (%)	Control	318.0 ± 17.7	341.4 ± 25.1
	AO	172.2 ± 22.5*	140.7 ± 28.9*
	mAO	278.2 ± 19.9^+^	254.2 ± 24.0^+^
	OR	279.2 ± 17.1	324.7 ± 29.2

Values are mean ± SEM.

For ∆Pes, Raw and CO_2_ response statistical differences were determined by a one-way analysis of variance. For room air ventilation and CO_2_ ventilation, statistical differences were determined by a two-way ANOVA followed by a post-hoc Student–Newman–Keuls test.

*∆Pes* inspiratory swings in esophageal pressure, *raw* airway resistance, *AO* obstructive, *mAO* mild obstruction, *OR* obstruction removal.

*p < 0.01—C vs. AO, mAO or.

^+^*p* < 0.05, mAO vs. AO.

**Figure 3:**
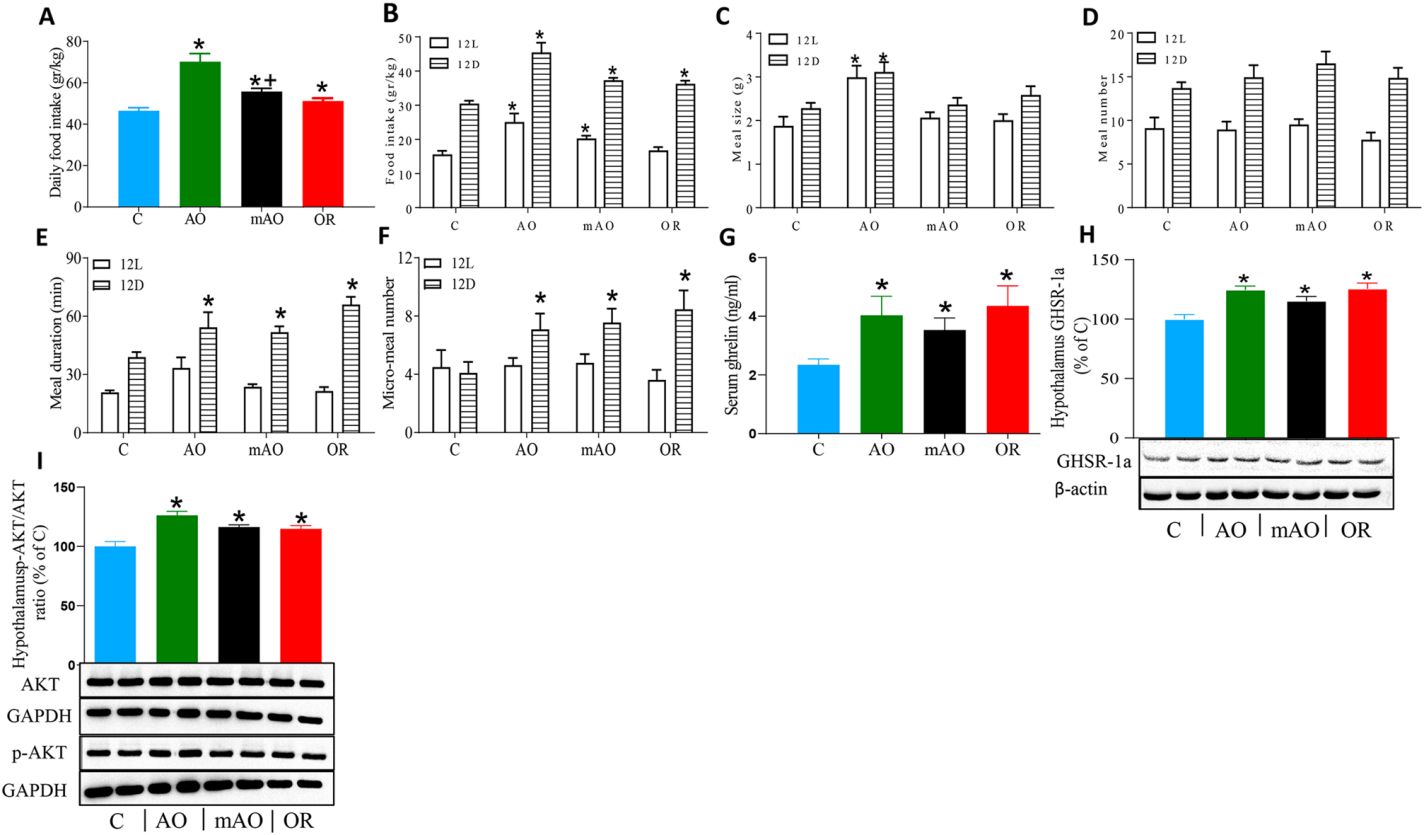
Energy intake and hormones: **(A)** daily food intake; **(B)** light/dark food intake; **(C)** meal size; **(D)** meal number; **(E)** meal duration; **(F)** micro-meal number; **(G)** serum ghrelin; **(H)** hypothalamic GHSR1a; **(I)** hypothalamic pAKT/AKT ratio; 12L—twelve hours lights-on period; 12D—twelve hours lights-off period (active period, 21:00–09:00); GHSR1a—growth hormone secretagogue receptor 1a; blue—control; green—obstructive; black—mild obstructive; red—obstruction removal; values are mean ± SEM. * *p* < 0.01, C vs. AO or mAO group. + *p* < 0.05, AO vs. mAO. In **(A,G–J)**, statistical differences were determined by a one-way ANOVA. In **(B–F)**, statistical differences were determined by a 2-way ANOVA, followed by a post-hoc Student–Newman–Keuls test.

Food intake increased by 50.9% (*p* < 0.01), 20% (*p* < 0.01), and 10.7% (*p* < 0.05) in the AO, mAO, and OR groups, respectively (*p* < 0.01, Fig. 3A, Table 2). Food intake in AO and mAO was elevated in light and dark phases, and only during the dark phase in the OR animals (*p* < 0.01, Fig. 3B). Meal size was elevated only in AO groups in light and dark phases (*p* < 0.01, Fig. 3C) and no significant changes were found in meal number (Fig. 3D). Increased feeding was related to elevated meal duration and the number of micro meals during the dark phase (*p* < 0.01, Fig. 3E,F). Elevated energy intake associated with up-regulation of serum ghrelin (*p* < 0.01, Fig. 3G) and GHSR1a (*p* < 0.01, Fig. 3H). The p-Akt/Akt ratio increased significantly (*p* < 0.01, Fig. 3I) by 25%, 16%, and 15% in the AO, mAO, and OR groups, respectively.

**Table 2:** Energy metabolism at week 10.

	Control	AO	mAO	OR
N	14	8	21	16
Food intake (g/kg)	46.4 ± 1.4	70.1 ± 3.9**	55.7 ± 1.5**^+^	51.1 ± 1.3*
Body mass index (g/cm^2^)	0.77 ± 0.01	0.62 ± 0.02**	0.74 ± 0.01^+^	0.76 ± 0.01
O_2_ consumption (ml/min/kg)	16.4 ± 0.3	20.9 ± 1.2**	18.3 ± 0.3*^+^	17.1 ± 0.3
CO_2_ production (ml/min/kg)	15.2 ± 0.2	19.2 ± 1.2**	16.8 ± 0.3*^+^	15.8 ± 0.3
EE (Kcal/h/kg)	4.9 ± 0.09	6.2 ± 0.38**	5.4 ± 0.1*^+^	5.1 ± 0.1
R_EE (Kcal/h/kg)	3.6 ± 0.07	4.5 ± 0.2**	4.0 ± 0.09*^+^	3.6 ± 0.1

Values are mean ± SEM over 24 h.

Statistical differences were determined by a one-way analysis of variance.

*EE* energy expenditure, *R_EE* resting energy expenditure, *AO *obstructive, *mAO* mild obstruction, *OR* obstruction removal.

*p < 0.05, **p < 0.01—C vs. AO, mAO or + *p* < 0.05 mAO vs. AO.

**Figure 4:**
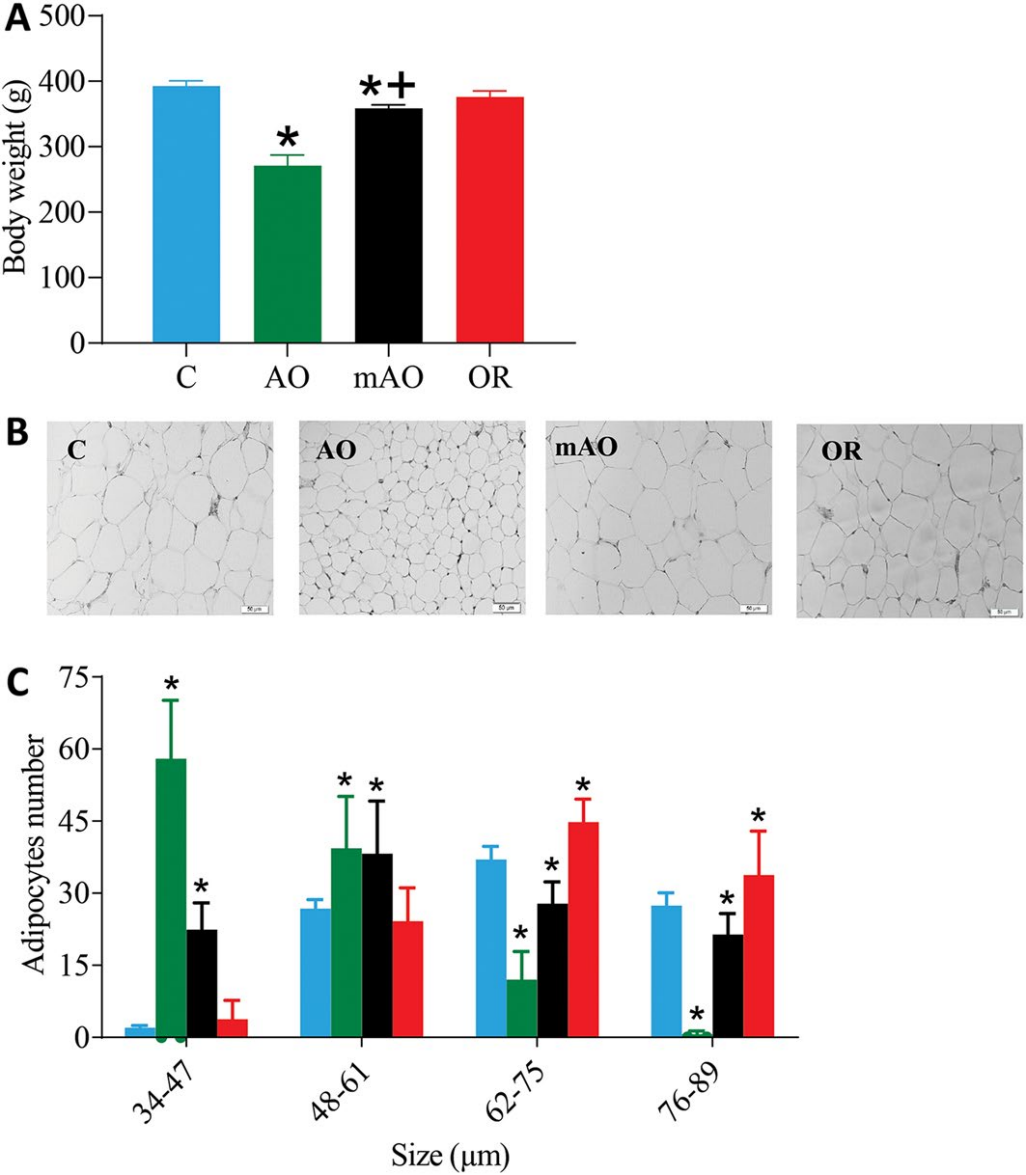
Retroperitoneal adipocyte analysis: **(A)** body weight; **(B)** histological images of retroperitoneal adipose tissue hematoxylin and eosin staining (magnification × 40); **(C)** adipocyte diameter; **(C)** frequency distribution of adipocyte size, bars represent the number of adipocytes; *C* control; *AO* obstruction; *mAO* mild obstruction; *OR* obstruction removal. Values are means ± SEM. * *p* < 0.01, C vs. AO or mAO group. In **(B)**, statistical differences were determined by a one-way ANOVA. In C, statistical differences were determined by two-way ANOVA, followed by a post-hoc Student–Newman–Keuls test.

**Figure 5:**
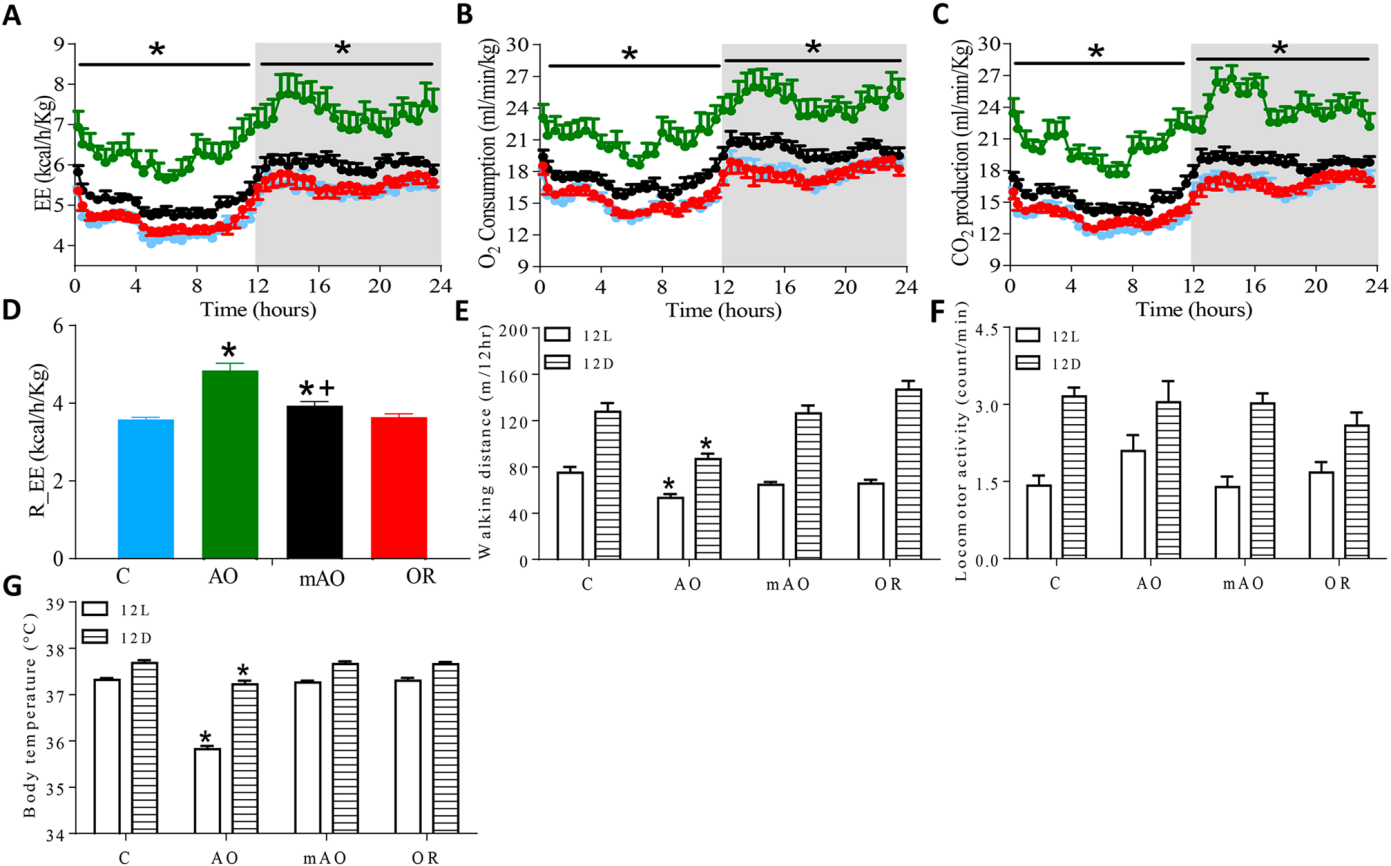
Energy metabolism analysis: **(A)** energy expenditure; **(B)** oxygen consumption; **(C)** carbon dioxide production; **(D)** resting energy expenditure; **(E)** walking distance; **(F)** locomotor activity; **(G)** body temperature. Gray area in **(A–C)** represents light of phase (active period, 21:00–09:00) on a 12:12-h cycle. Values in **(A–D)** were adjusted to effective body mass by ANCOVA analysis. 12L—12 h lights-on period; 12D—12 h lights-off period; EE—energy expenditure; R_EE—resting EE calculated as mean value for 30 min period with lowest EE; O_2_—oxygen; CO_2_—carbon dioxide; MA—locomotion activity; blue—control; green—obstructive; black—mild obstructive; red—obstruction removal; values are mean ± SEM. * *p* < 0.01, C vs. AO group or mAO group. + *p* < 0.05, AO vs. mAO. In **(A–C)** and **(E–G)**, statistical differences were determined by a two-way ANOVA, followed by a post-hoc Student–Newman–Keuls test. In **(D)**, statistical differences were determined by a one-way ANOVA.

**Figure 6:**
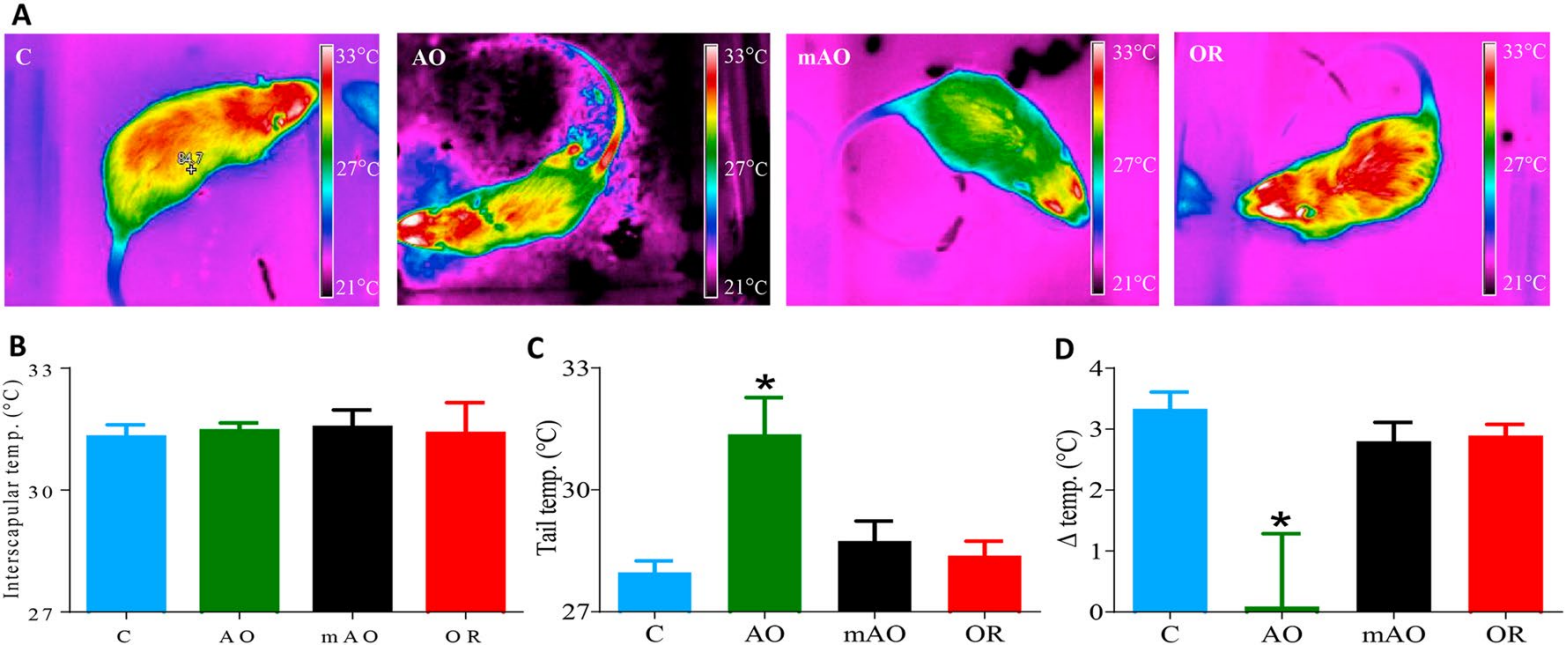
Thermography analysis: **(A)** Representative infrared images. **(B)** Interscapular temperature; **(C)** tail temperature measured 0.5 cm from the tail base; **(D)** the differences between interscapular and tail temperatures; blue—control; green—obstructive; black—mild obstructive; red—obstruction removal. * *p* < 0.01, C vs. AO or mAO group. In **(D)**, statistical differences were determined by a one-way ANOVA.

Body mass index (BMI) decreased by 19.5% and 4% in AO and mAO animals, respectively (*p* < 0.01; Table 2). The BMI of the OR group was similar to the control group. Reduced body weight in the AO and mAO groups (Fig. 4A) was associated with a reduced retroperitoneal adiposity cell distribution diameter (*p* < 0.01, Fig. 4B). A trend of increased adiposity cell size distribution was found in the OR group (*p* < 0.01, Fig. 4C). Total energy demand increased by 26.5% and 10.2% in the AO and mAO groups, respectively (*p* < 0.01, Fig. 5A, Table 2). Increased energy demand was associated with the elevation of O_2_ consumption and CO_2_ production (*p* < 0.01, Fig. 5B,C, Table 2). Following OR, energy demand was normalized to the values of the control group (Fig. 5A,D). Walking distance and body departure significantly decreased only in the AO group (Fig. 5E,G). No significant changes were found in locomotor activity in any of the groups (Fig. 5F). Infrared thermography of interscapular and tail surface temperatures in the mAO and OR groups were similar to controls (Fig. 6A,B). Tail surface in the AO group increased by 3 °C (*p* < 0.01, Fig. 6C), and temperature differences between the interscapular area and the tail was close to zero (*p* < 0.01, Fig. 6D).

## Discussion

We found that mAO vs. AO animals can consume a sufficient amount energy to maintain respiratory homeostasis and maintain thermoregulation. Increased feeding in the OR group was associated with largely irreversible feeding associated with increased serum ghrelin, hypothalamic GHSR1α, and p-AKT/Akt ratio, despite normalization of breathing and energy requirements. Our study supports the need for lifestyle eating behavior management, in addition to endocrine support, in order to attain healthy metabolic physiology in OSA patients.

Several models were used to explore the effects of OSA on the functional consequences of sleep, metabolism, and diurnal behavior^27,28^. In the current study, we explored the effects of mild AO on feeding behavior. We achieved mAO by generating a larger trachea diameter at the site of obstruction relative to the AO group. Both obstructed groups exhibited up-regulation of energy expenditure that was associated with the increased work of breathing. However, the magnitude of increased energy expenditure was about 70% less in the mAO animals, indicating less severe airway obstruction in this group. The mAO model is more comparable to human OSA, which is associated with metabolic abnormalities without evidence of energy crisis^1–3,7,8,10^. In both obstructed groups, increased feeding was associated with the up-regulation of serum ghrelin, hypothalamic GHSR1a, and the p-Akt/Akt ratio. However, mAO animals were able to consume a sufficient amount energy to meet additional energy requirements of airway loading and maintain healthy metabolic physiology. AO animals, on the other hand, could not maintain healthy metabolic physiology and thermoregulate despite a 50% elevation of energy intake. This loss of thermoregulation was probably associated with non-functioning brown adipose tissue^18^ and heat loss via the tail. In our study both AO and mAO exhibited increased feeding associated with an elevation in gut-derived ghrelin. Previously, we found that AO animals had considerable sleep fragmentation and were awake 20% more during the 12-h lights-on period^16,17^. Short sleep per se may stimulate the secretion of ghrelin and feeding^29^ by activation of GHSR1a^30^. Increased feeding in the OR group was associated with largely irreversible increased feeding hormones even though breathing and energy requirements were normalized. It is not clear why treatment of OSA does not restore healthy metabolic physiology and predisposes accelerated weight gain^2–9^. Our study suggests that feeding hormones and imbalance of GHSR1a and Akt pathway participate in this persistent elevation of feeding behavior following OR; that in the long run may lead to obesity, insulin resistance and type 2 diabetes^21–23^. Weight gain following OSA treatment was not associated with changes in physical activity or sleep quality^6^, highlighting the need for lifestyle modifications in order to prevent weight gain^6,8^.

In the current study, increased ventilation in obstructed groups was a physiological response proportional to the magnitude of increased CO_2_ production in order to maintain respiratory homeostasis. Chronic ventilatory load may diminish sensitivity to CO_2_, and the decreased response to CO_2_ in our AO, but not mAO animals, accompanies many diseases associated with chronic hypercapnia. Arterial blood gases and serum lactate were in the normal range, suggesting that animals in all groups maintained ventilation and pO_2_. Moreover, AMPK participates importantly in regulating the tissue AMP/ATP ratio and may become activated by hypercapnia and lactic acidosis^24–26^. We did not observe significant changes in muscle AMPK phosphorylation in our animals. Exposure to hypercapnia can increase AMPK phosphorylation and mediate skeletal muscle protein degradation and loss of body weight^24,25,27^. Our study suggests that AMPK did not play a role in weight regulation of these rats during obstruction or OR. It is possible that slow body weight gain was associated with a reduction in adiposity tissue mass^16,18,28^. In calorie restriction, decreased body weight was associated with organ weight losses^31^. It is possible that the increased resistance produced in our animals may lead to hyperinflation and an alteration in functional residual volume with an adverse effect on diaphragm contractility and CO_2_ sensitivity. In AO animals, however, we found an increase in diaphragm mass and contractility with no change in muscle length or contractility^28^, while others found increased endurance^32^.

In both groups of obstructed rats, we found an increase in total and resting energy expenditure during the day. Estimates of the energy expenditure exchange of O_2_ with CO_2_ are valid if metabolic energy is calculated from aerobic sources^33,34^. In our study, the impressive elevation of energy expenditure found in AO vs. mAO animals was associated with the degree of airway obstruction. During quiet breathing, oxygen consumption of the respiratory muscles is less than 2% of resting VO_2._ The cost of breathing increases robustly during resistive breathing and not by isocapnic hyperventilation^35^. However, experimentally increased resistive breathing by a verity of diseases can significantly increase the energetic breathing cost. Although AO is not sleep related^15^, it is unlikely that this explains the higher energy demand found in AO. Nevertheless, there are several similarities between the AO model to OSA^36^: AO is associated with partial sleep loss and sleep fragmentation^15^ and sleep loss in rodents can lead to loss of body weight and thermoregulation despite an increased energy intake^37,38^. Resting metabolic rate was significantly higher in adult OSA patients^14,39,40^, and correlates with increased OSA severity^41,42^, and earlier studies in children with OSA demonstrated that energy expenditure during sleep correlates with the increased work of breathing^43^. However, in this model, in contrast to OSA, increased airway resistance was both inspiratory and expiratory and not exclusively sleep-related, and it resembled conditions such as increased nasal or subglottic resistance, tracheal stenosis, etc. In OSA however, increased airway resistance is sleep-related with diurnal recovery^1,44^.

## Conclusion

Mild AO vs. AO animals were able to consume sufficient amounts of energy to maintain respiratory and healthy metabolic physiology. Obstruction removal was associated with largely irreversible increased feeding despite the normalization of breathing and energy requirements. Up-regulation of feeding associated with elevated serum ghrelin, and with hypothalamic GHSR1a and the p-Akt/Akt ratio. Our study supports the need for lifestyle management of eating behavior, in addition to endocrine support in order to attain healthy metabolic physiology in OSA patients.

## Methods

### Animals

This study was approved by the Ben-Gurion University of the Negev Animal Use and Care Committee, protocol number IL-40-07-2018. All protocols comply with American Physiological Society Guidelines and study is reported in accordance with Animal Research: Reporting of In Vivo Experiments (ARRIVE) guidelines.

### Upper airway obstruction

Rats were anesthetized using tribromoethanol (200 mg/kg i.p.). Upper airway obstruction (AO), mild airway obstruction (mAO), or sham control surgeries were performed in 22-day-old (48–55 g) male Sprague–Dawley rats (Fig. 2) (see Supplementary Methods)^18,20^.

Both AO and mAO groups underwent similar surgery in which a silicon band 0.5 cm long was placed around the trachea, and two sutures were looped around it and tightened to constrict its diameter. Based on the increased inspiratory esophageal pressure swings, we established two levels of obstruction. The degree of trachea obstruction was confirmed by histology after animals were sacrificed. On day 14 (14 days), obstruction removal (OR) surgery was performed on mAO animals (Fig. 1)^17,18,20^. The mortality rates of all surgical procedures were less than 10%. Animals were kept on a 12–12 light–dark cycle with lights on at 09:00 at 231.0 ºC. Food (3272 kcal/kg) and water were given ad libitum*.*

### Experimental schedule

Surgery (trachea obstruction or sham) was performed and animals were returned to their home cages (Fig. 2). On day 14, OR surgery was performed on 16 animals, whereas all other animals underwent repeated sham surgery. Measurements for all groups were performed at the same time: respiration samplings were performed on day 35 (35 days) and on day 66 (66 days) by whole body plethysmographing. Recording body temperature and locomotion activity was performed on day 68 for 24 h. Metabolic and activity profiles were performed on days 69–72. Shortly before animals were sacrificed on day 73, they were anesthetized, BMI was calculated, and serum and tissues were extracted. Arterial blood gases, serum ghrelin, lactate, hypothalamic GHSR-1a and the p-Akt/Akt ratio were determined for all groups 1–3 h after lights on.

### Respiratory and energy metabolism

Inspiratory swings in esophageal pressure (∆Pes) were measured in anesthetized animals prior to plethysmography measurement, and airway resistance was calculated as ∆Pes/inspiratory flow^18^. Respiratory activity was recorded by plethysmography (Buxco, DSI, St. Paul, MN, USA), as previously described (see Supplementary Methods). Trachea histology was determined, as previously described^17–19^.

Interscapulum and tail surface temperatures were measured in conscious animals at 27 °C using an infrared camera (Fluke, Everett, WA, USA)^18,33^. In a subset of n = 5 animals in each group, body temperature (Tb) (± 0.1 °C) and locomotion activity (MA) were analyzed by a free-floating telemetric transmitter (model TA11TA-F10, DSI, St. Paul, MN, USA) using the Dataquest A.R.T. system (DSI, St. Paul, MN, USA)^14,16,19^.

Metabolic activity was measured using a Sable Instruments system (Sable Instruments, Las Vegas, NV, USA), as previously described (see Supplementary Methods)^18,20^. Animals were allowed a 24–48 h acclimation period followed by a 48-h sampling duration. Effective body mass was calculated by ANCOVA analysis^18,34^. Energy expenditure was calculated as VO_2_ × (3.815 + 1.232 × respiratory quotient), and was normalized to effective body mass. Resting energy expenditure was calculated as the mean value for a 30-min period with the lowest energy expenditure. The respiratory quotient was calculated as the ratio of the CO_2_ produced by the O_2_ consumed.

### Protein analysis

At sacrifice, 2–3 h after lights on, the serum was used for measurement of ghrelin and lactate, using a specific enzyme-linked immunosorbent assay kit^15–20^. The low and high detection limits for ghrelin were 0.04 and 10 ng/mL (kit number EZRGRA-90K; Merck Millipore, Rosh Haayin, Israel), and the intra- and inter-assay CVs were 1.1% and 3.2%, respectively. Serum lactate was determined at the Soroka University Medical Center Biochemistry laboratory. In a subset of n = 4 of control animals, AO, and OR arterial blood gases (pH, PCO_2_, PO_2_, and HCO_3_^−^) were determined in parallel to their serum lactate level on day 66 after surgery. Antibodies used for evaluation of the hypothalamic protein extract by western immunoblot analysis (see Supplementary Methods) were GHSR1a (Santa Cruz Biotechnology, Santa Cruz, CA, USA), Akt and p-Akt (Cell Signaling Technology, Danvers, MA, USA), soleus AMPK and p-AMPK (MP Biomedical Solon, OH, USA), and GAPDH (Proteintech, Rosemont, IL).

### Data analysis

Significance between groups was determined by a one-way analysis of variance. A two-way analysis of variance for repeated measures or plethysmography was used to determine the significance between time and group. Post-hoc analysis was performed by a Student–Newman–Keuls test. Null hypotheses were rejected at the 5% level.

## Supplementary Information

Supplementary Information.
