# Supplementary material for: Changes in energy metabolism and respiration in different tracheal narrowing in rats
Source: Sci Rep. 2021 Sep 27;11:19166. doi: 10.1038/s41598-021-98799-8 (PMC8476542; doi:10.1038/s41598-021-98799-8)
Supplement: Supplementary file 1 — Supplementary Information. [file 41598_2021_98799_MOESM1_ESM.pdf]

Supplementary Information

Changes in Energy Metabolism and Respiration in Different Tracheal Narrowing in Rats

Yael Segev, Haiat Nujedat, Eden Arazi, Mohammad H. Assadi, and Ariel Tarasiuk

Supplement results

During the observation period, animals maintained their normal arterial blood gases (**Supplementary Table 1**) and serum lactate level (**Supplementary Figure 1**).

Supplementary Table 1: Arterial blood gases

	Control	AO	OR
PO_2 (mmHg)	102.5±7.6	107.58±9.1	90.5±6.9
PCO_2 (mmHg)	45.68±6.8	44.55±4.7	42.85±6.2
pH (units)	7.36±0.05	7.36±0.04	7.38±0.03
HCO_3^- (mEq/L)	25.88±1.6	24.35±1.2	24.8±2.3

PO_2 – arterial O_2 pressure; PCO_2 – arterial CO_2 pressure; pH – arterial pH; HCO_3 – calculated arterial bicarbonate; Values are mean ± SD.

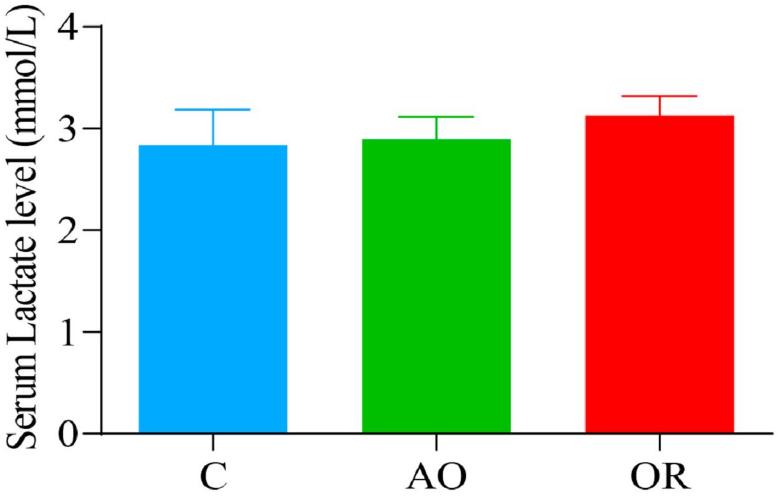

Supplementary Figure 1: Serum lactate level. C – control; AO – obstructive; mAO – mild obstruction; OR – obstruction removal. Blue – control; Green – obstructive; Red – obstruction removal.

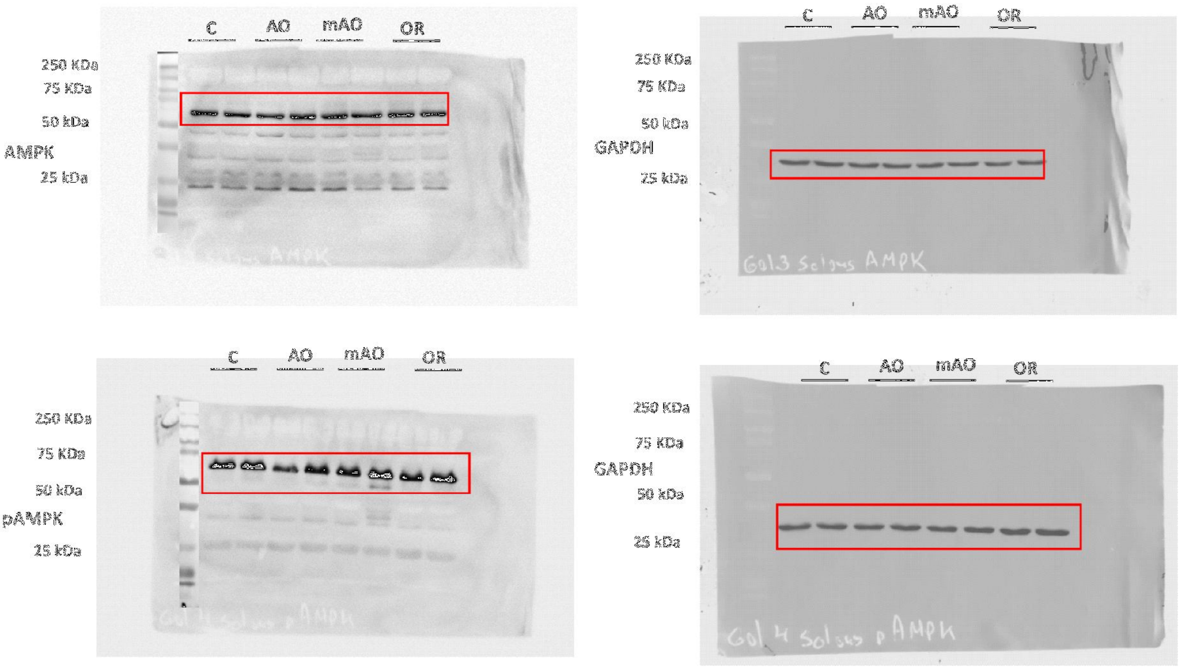

Supplementary Figure 2: Full-length un-cropped original western blots for AMPK and p-AMPK referred to in Figure 2I. Post transfers membrane was cut beyond marker range in order to fit within hybridisation box. Blots were imaged on MicroChemi 4.2 (DNR, Bioimaging Systems, Jerusalem, Israel).

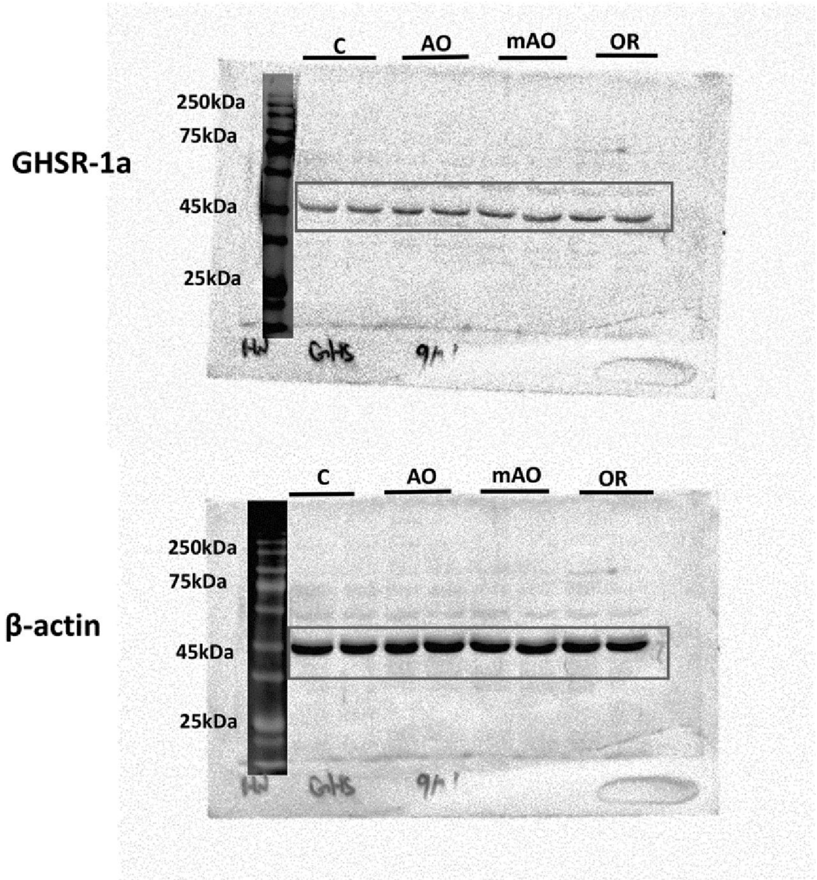

<u>Supplement Figure 3</u>: Full-length un-cropped western blots for GHSR1a referred to in Figure 3H. Post transfers membrane was cut beyond marker range in order to fit within hybridisation box. Blots were imaged on MicroChemi 4.2 (DNR, Bioimaging Systems, Jerusalem, Israel).

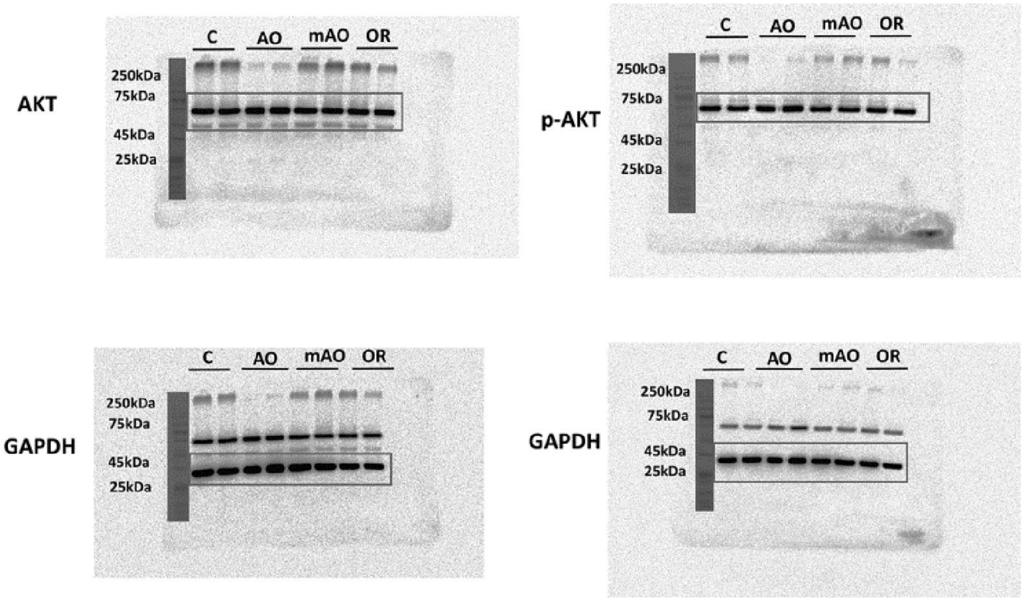

<u>Supplement Figure 4</u>: Full-length un-cropped western blots for AKT and p-AKT referred to in Figure 3I. Post transfers membrane was cut beyond marker range in order to fit within hybridisation box. Blots were imaged on MicroChemi 4.2 (DNR, Bioimaging Systems, Jerusalem, Israel).

Supplementary Methods

Animals

This study was approved by the Ben-Gurion University of the Negev Animal Use and Care Committee, protocol number IL-40-07-2018. All protocols comply with the American Physiological Society Guidelines. Male 22-day-old Sprague-Dawley rats (48–55 gr) were used. Animals were kept on a 12–12 light-dark cycle with lights on at 09:00 at 23±1.0?C. Animals were given food and water *ad libitum.*

Surgery

Tracheal narrowing surgery (anesthesia tribromoethanol, 200 mg/kg i.p., Sigma Aldrich Ltd., Israel) was used to induce upper airway obstruction (AO) or mild airway obstruction (mAO) in 22-day-old male Sprague-Dawley rats (day 0, Figure 1)[1-4] A circumferential silicon band 0.5 cm long was placed around the trachea to induce tracheal-narrowing. Two sutures were looped around the band and tightened, thus, constricting the trachea so as to increase inspiratory esophageal pressure swings. Controls underwent sham surgery with no tracheal narrowing. On day 14, the mAO was divided, and obstruction removal (OR) of the silicon band was performed; the other groups underwent repeat sham surgery. On day 61 (61 d), a free-floating telemetric transmitter (model TA11TA-F10, DSI, St. Paul, MN, USA) was inserted into the abdominal cavity. The transmitter was not attached to the peritoneum and was able to move freely among the peritoneal organs.[5,6] Following surgery, prophylactic enrofloxacin 5 mg/ml (s.c.) and water containing ibuprofen (0.1 mg/ml) were given for three days. [1,2]

Telemetry recordings

On 68 d, body temperature (±0.1°C) and MA were continuously monitored using the Dataquest A.R.T. system (DSI, St. Paul, MN, USA). MA (counts/min) is obtained by counting the number of impulses per minute detected by changes in signal strength. The signal is received by an antenna under each animal's cage and transferred to a peripheral processor connected to a personal computer. All transmitters were calibrated both before surgery and at the completion of the experiment to ensure validity of the biotelemetry measurements. Tb and MA raw data were collected at 1-sec intervals in unrestrained rats. Raw Tb and MA data are graphically presented as 1-h averages for ease of presentation, [1,2,5]

Respiratory measurements

We used unrestrained whole-body plethysmography (Buxco, DSI, St. Paul, MN, USA) to evaluate the respiratory rate (b/min), tidal volume (ml/100 gr) and minute ventilating, as previously described. Data were analyzed by FinePointe software (Buxco Research Systems, V. 2.4.4.9064).[2] Following acclimatization to the chamber, respiratory measurements were performed at 66–67 d in room-air breathing and also following exposure to a gas mixture of 7% CO_2/93% O_2.

Metabolic and activity profiles

Metabolic and activity profiles of the rats were measured using the Promethion High-Definition Behavioral Phenotyping System (Sable Instruments, Inc., Las Vegas, NV, USA).[2] Data acquisition and instrument control were performed using MetaScreen software V.2.3.14.6, and the obtained raw data were processed with ExpeData version 1.9.14 using an analysis script detailing all aspects of data transformation. Respiratory

gases were measured by the GA-3 gas analyzer (Sable Systems, Las Vegas, NV, USA) using a pull-mode, negative-pressure system. Air flow was measured and controlled by FR-8, with a set flow rate of 2,500 ml/min. Water vapor was continuously measured, and its dilution effect on O_2 and CO_2 was mathematically calculated. Animals had free access to food and water, and were allowed a 24-h acclimation period followed by a 48-h sampling duration. Effective mass was calculated by ANCOVA analysis, as described previously.[2,7] The respiratory quotient (RQ) was calculated as the ratio between CO_2 produced to O_2 consumed. Resting EE was defined as the mean value for the 30-min period with the lowest EE (see Methods section). Food and water intake were normalized to effective body mass. The interscapular and tail surface temperatures were measured in conscious animals at 27°C using a Fluke Ti400 infrared camera (Fluke, Everett, WA, USA).[2] The body mass index was calculated by dividing body weight (grams) by the body length (cm) squared.

Blood collection

In a subset n=4 of control animals, AO and OR arterial blood gases and serum lactate were determined at 66 d after surgery. Blood gas samples were drawn in a pre-heparinized syringe, placed on ice, and immediately analyzed on a blood gas analyzer (RAPID Point 500, Siemens, Erlangen, Germany). Plasma ghrelin was determined by specific enzyme-linked immunosorbent assay (ELISA) kits, used according to the manufacturer's instructions. Blood was drawn, placed in a microcentrifuge tube on ice, and specimens were centrifuged cold (2,000 *g* for 10 min). The plasma was pipetted into chilled microcentrifuge tubes for subsequent storage at -80°C. The low

and high detection limits for ghrelin were 0.04 and 10 ng/mL (Merck Millipore, Rosh Haayin, Israel); the intra- and inter-assay CVs were 1.1% and 3.2%, respectively.

Tissues harvested

At the conclusion of the study, the hypothalamus and soleus muscles were dissected, frozen in liquid nitrogen, and stored at -80°C.

Western immunoblot analysis

The hypothalamus and soleus were homogenized on ice with a polytron (Kinetica, Littau, Switzerland) in lysis buffer (50mM Tris, pH 7.4, 0.2% Triton X-100) containing 20mM sodium pyrophosphate, 100mM NaF, 4mM EGTA, 4mM Na3VO4, 2mM PMSF, 0.25% aprotinin, and 0.02 mg/ml leupeptin. Extracts were centrifuged for 20 min at 13,000 rpm at 4°C, and the supernatants were collected and frozen. Homogenates were mixed with a 4^{a} sample buffer and boiled for 5 min. Then 100 -μg portions of sample proteins were loaded in each gel lane and subjected to 7.5–15% SDS polyacrylamide gel, and electroblotted into nitrocellulose membranes. Blots were blocked for 1 h in TBST (0.05% Twin-20) buffer (10mM Tris, pH 7.4, 138mM NaCl) containing 5% non-fat dehydrated milk, followed by overnight incubation with UCP1 antibodies (Abcam Cambridge, MA, USA) and β-actin (MP Biomedicals, Solon, OH, USA). After washing three times for 15 min in TBST (0.05% Twin-20), the blots were incubated with secondary anti-mouse (β-actin) or anti-rabbit UCP1 antibodies conjugated to horseradish peroxidase for 1 h at room temperature, and then washed again three times. The band antibody was visualized by enhanced chemiluminescence (ECL; Biological Industries, Beit Haemek, Israel), and images were taken using

Microchemi 4.2 (DNR Bio Imaging System, Jerusalem, Israel). Densitometric analyses were performed using Image J.[1,2,4,5]

Trachea Histology

Tracheal segments were fixed in 4% formalin for 48 h at room temperature. After fixation, the silicon band was removed from the tracheal segments of all obstructed rats. All tracheal segments were then embedded in paraffin, and sections were cut (5 µm thick) and collected on Superfrost™ Plus slides for histology staining with hematoxylin and eosin.[1,2] Photomicrographs were obtained by a light microscope (Olympus BX41, Japan) at [a]20 magnification (Zeiss Axioplan MR12, Germany) equipped with a digital camera (Olympus DP72) connected to a PC that used tissue histology morphometric software (cellSens Entry Imaging Software, Olympus Corp., Tokyo, Japan). The internal border of the trachea was outlined, and its diameter was measured for each animal.

Supplement References

1. Assadi, M.H., Shknevsky, E., Segev, Y., & Tarasiuk A. Abnormal growth and feeding behavior persist after removal of upper airway obstruction in juvenile rats. *Sci. Rep.* **7,** 2730 (2017).
2. Assadi, M.H., Segev, Y., & Tarasiuk. A. Irreversible metabolic abnormalities following chronic upper airway loading. *Sleep* **24,** zsz176 (2019).
3. Tarasiuk, A., Scharf, S.M., & Miller, M.J. Effects of chronic resistive loading on inspiratory muscles in rats. *J. Appl. Physiol.* **70,** 216-222 (1991).

4. Tarasiuk, A., Levi, A., Berdugo-Boura, N., Yahalom, A., & Segev, Y. Role of orexin in respiratory and sleep homeostasis during upper airway obstruction in rats. *Sleep* **37,** 987-998 (2014).
5. Segev, Y., Berdugo-Boura, N., Porati, O., & Tarasiuk, A. Upper airway loading induces growth retardation and changes in local chondrocyte IGF-1 expression is reversed by stimulation of GH release in juvenile rats. *J. Appl. Physiol.* 2008; **105** (2-3): 1602-1609.
6. Leon, L.R., Walker, L.D., DuBose, D.A., & Stephenson LA. Biotelemetry transmitter implantation in rodents: impact on growth and circadian rhythms. *Am. J. Physiol. Regul. Integr. Comp. Physiol.* **286,** R967-R974 (2004).
7. Tsch?p, M.H. A guide to analysis of mouse energy metabolism. *Nat. Methods* **9,** 57-63 (2011).
